# Field-relevant doses of the systemic insecticide fipronil and fungicide pyraclostrobin impair mandibular and hypopharyngeal glands in nurse honeybees (*Apis mellifera*)

**DOI:** 10.1038/s41598-017-15581-5

**Published:** 2017-11-09

**Authors:** Rodrigo Zaluski, Luis Antonio Justulin, Ricardo de Oliveira Orsi

**Affiliations:** 1 Núcleo de Ensino, Ciência e Tecnologia em Apicultura Racional (NECTAR), São Paulo State University (UNESP), School of Veterinary Medicine and Animal Science, Department of Animal Production, Botucatu, SP Brazil; 2 São Paulo State University (UNESP), Institute of Biosciences, Department of Morphology, Botucatu, SP Brazil

## Abstract

Global decreases in bee populations emphasize the importance of assessing how environmental stressors affect colony maintenance, especially considering the extreme task specialization observed in honeybee societies. Royal jelly, a protein secretion essential to colony nutrition, is produced by nurse honeybees, and development of bee mandibular glands, which comprise a reservoir surrounded by secretory cells and hypopharyngeal glands that are shaped by acini, is directly associated with production of this secretion. Here, we examined individual and combined effects of the systemic fungicide pyraclostrobin and insecticide fipronil in field-relevant doses (850 and 2.5 ppb, respectively) on mandibular and hypopharyngeal glands in nurse honeybees. Six days of pesticide treatment decreased secretory cell height in mandibular glands. When pyraclostrobin and fipronil were combined, the reservoir volume in mandibular glands also decreased. The total number of acini in hypopharyngeal glands was not affected, but pesticide treatment reduced the number of larger acini while increasing smaller acini. These morphological impairments appeared to reduce royal jelly secretion by nurse honeybees and consequently hampered colony maintenance. Overall, pesticide exposure in doses close to those experienced by bees in the field impaired brood-food glands in nurse honeybees, a change that could negatively influence development, survival, and colony maintenance.

## Introduction

Pollination is an indispensable process that ensures ecosystem maintenance, plant reproduction, agriculture, and food security^1^. Approximately 35% of globally important food crops depend on pollinators^2^. Among the most common are honeybees (*Apis mellifera* L.), managed worldwide for pollination services^1,2^ and which generate considerable revenue in the beekeeping industry through their production of honey, pollen, propolis, beeswax, royal jelly, and apitoxin.

Despite the importance of honeybees and other pollinators, their global populations have seen major reductions^1,3–5^. The main factors responsible are anthropogenic actions that reduce and fragment pollinator habitat, affecting resource availability, the spread of diseases and parasites, and invasive species as well as pesticide exposure. These factors can act alone or synergistically to impair pollinator maintenance^5,6^.

In particular, existing concerns about pesticide exposure to non-target organisms have raised questions regarding whether field-relevant doses are harmful to honeybees^7–9^. High pesticide concentrations can immediately kill off colonies^10^, while chronic exposure to sub-lethal doses, which occurs frequently^3,10–12^, may have delayed negative effects^13^. These sub-lethal effects include compromised resource collection; behavioural changes; decreased longevity, immune function, population growth, reproduction, and learning performance; and the creation of new queens, thus, ultimately influencing colony survival^5,13–21^. Specifically, pesticide or xenobiotic exposure is linked to midgut-cell impairment^22–26^ that can reduce nutrient digestion and absorption in insects^27,28^ and potentially damage honeybee physiology.

Insecticides are often related to honeybee losses because of their high toxicity and lack of specificity^15,20,29^. However, pollinators are more likely to encounter fungicides during foraging^13^. Because fungicides are traditionally considered safe to pollinators, they can be applied on blooming crops, increasing non-target exposure^13,30^ and are becoming the most commonly found pesticide in bee colony combs and food stores^11,31^. Unfortunately, studies show that fungicides do exert a negative effect on bees. Fungicide exposure causes impaired ATP production, increased virus titres, poor brood rearing and queen emergence, population declines, and higher disease susceptibility^13,32,33^. Fungicide exposure can also cause nutritional deficiencies in colonies, resulting in similar symptoms to those of malnutrition even when pollen is available^13^.

Honeybees are eusocial insects living in highly organized societies. As ‘super-organisms’, bee colonies are maintained through specialized tasks performed by individuals of a particular caste^34^; thus, the entire colony is affected if exposure to toxicity hampers a single task^35^. One of the worker specializations is the nurse, which is characterized by pheromone-stimulated^36,37^ development of mandibular and hypopharyngeal (or brood-food) glands in the head (at ~6 d old)^37,38^. Through the secretion of proteinaceous compounds^35^ and pheromones, such as 10-hydroxy-dec-2-enoic acid^38,39^, by these glands, nurse bees produce royal jelly after consuming high quantities of pollen^36,37,40^. As other colony members have limited pollen-digesting capacity, royal jelly is their main source of protein^41^ and is used to feed young larvae, adult bees, and the queen^42,43^. Thus, a hive’s contact with pesticides would primarily occur through nurse bees that consume contaminated pollen, while the nurses themselves may be more susceptible to those pesticides^44,45^. However, few reports have characterized the impacts of the combination of different classes of pesticides in brood-food glands of honeybees.

Pyraclostrobin is a strobilurin, which is a group of systemic fungicides that inhibit mitochondrial respiration^46^; it is widely detected in the pollen of treated crops and in honeybee colonies^31,47–49^. Fipronil is a phenylpyrazole, a systemic neurotoxic insecticide that is widely used in agricultural and veterinary applications. Similar to pyraclostrobin, fipronil is detected in crop pollen and honeybee colonies^11,28,50,51^; it is also highly toxic to the latter^20^. Based on existing data, we hypothesized that exposure to these two pesticides in nurse honeybees would promote alterations to mandibular and hypopharyngeal glands, possibly through impairing nutrient digestion and absorption.

To investigate this hypothesis, newly emerged bees were chronically exposed to both pyraclostrobin and fipronil and then the condition of their brood-food glands was evaluated. Mandibular glands comprise a reservoir surrounded by secretory cells that form a pseudo-epithelium^38^, and hypopharyngeal glands are clusters of 8–12 secretory cells connected to the gland’s main channel^36^. Specifically, we examined secretory-cell height and reservoir volume of the mandibular glands as well as acini size and number in the hypopharyngeal glands. Cell height of the mandibular glands is a main indicator of development and secretory activity of these glands^38,52^, whereas acini size in hypopharyngeal glands indicates activity and the amount of royal jelly secreted^36,38,40,53,54^.

To evaluate the effects of pyraclostrobin and fipronil in doses as close as possible to those to which bees are exposed in the field during pollen collection, we chose a pyraclostrobin dose of 850 ppb, which is between the mean concentrations detected by Pettis *et al*.^31^ and Yoder *et al*.^47^ in pollen and beebread. For fipronil, we choose a dose of 2.5 ppb, which is close to the mean dose detected in pollen^29,50,51^.

Our results will provide insight on how damage to brood-food glands in nurse honeybees impacts colony maintenance. Consequently, this study will contribute to efforts aimed at addressing honeybee population declines through improved management of pesticide application.

## Results

### Individual and combined effects of pyraclostrobin and fipronil on mandibular glands

**Figure 1:**
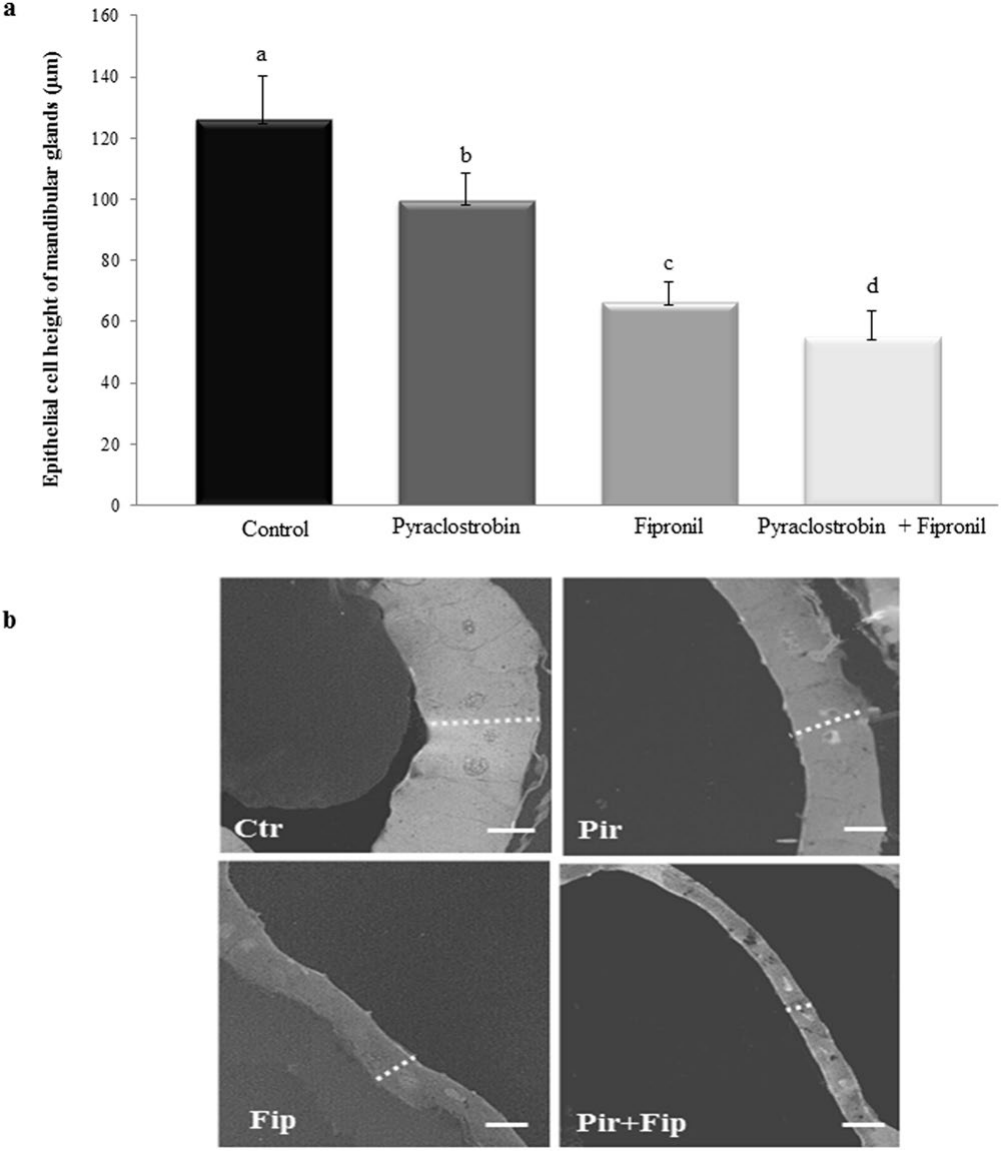
Effects of individual and combined exposure to pyraclostrobin and fipronil on epithelial cell height of mandibular glands in nurse honeybees. (**a**) Data from 10 histological sections of 10 randomly selected mandibular-gland cells for 10 bees (n = 1000 cells). Different letters over the bars represent statistically different groups (Kruskal–Wallis one-way ANOVA on ranks with Dunn’s post-hoc test for pairwise comparisons, p < 0.001). All data are represented as the median with interquartile range. (**b**) Representative histological sections showing mandibular gland epithelial cell height. Ctr: Control; Pir: Pyraclostrobin, Fip: Fipronil; Pir + Fip: Pyraclostrobin + Fipronil. Scale bar: 50 µm.

In the mandibular glands of pesticide-treated nurses, epithelial secretory cells were significantly decreased in height. Respectively, bees exposed to pyraclostrobin, fipronil, and pyraclostrobin + fipronil saw mean cell-height reduction by 21, 46, and 56%, compared with the control group (Fig. 1). Our results demonstrated that effects of the combination of pesticides on cell height were greater than those produced by exposure to either pesticide alone and indicated an additive effect.

**Figure 2:**
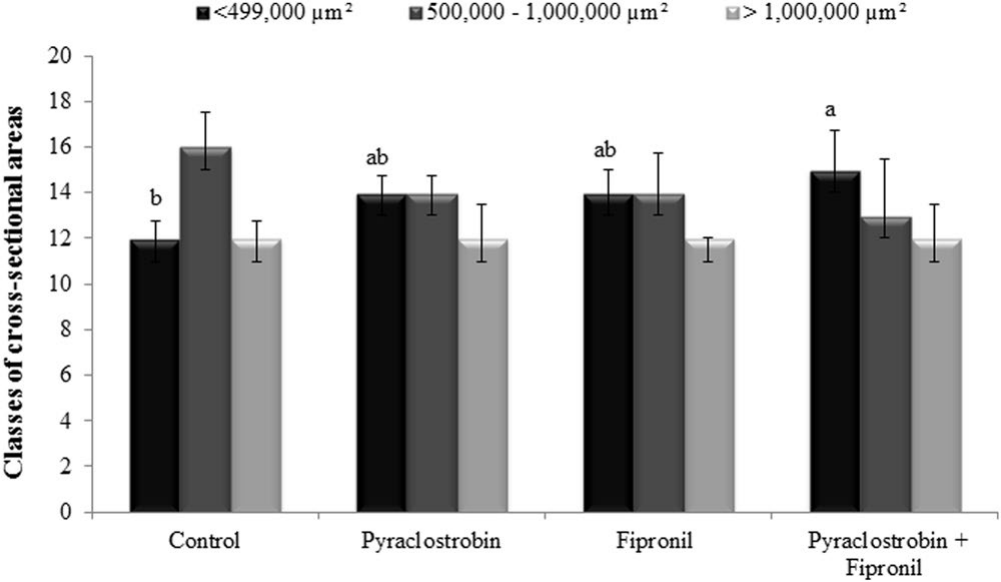
Classes of cross-sectional areas in the mandibular glands of nurse honeybees exposed to treatments of pyraclostrobin and fipronil both individually and combined. The total area (µm²) of serially collected histological sections (from mandibular gland pairs of 10 honeybees) taken at 30 µm intervals (20 sections/bee) were measured and categorized based on their areas 6 d after exposure. Different letters over the bars represent statistically different groups (Kruskal–Wallis one-way ANOVA on ranks with Dunn’s post-hoc test for pairwise comparisons, p = 0.003). All data are represented as the median with interquartile range.

Combined pyraclostrobin + fipronil exposure significantly affected the reservoir area of mandibular glands. Small cross-sectional areas (<499,000 µm²) were more present under combined exposure than in the control, demonstrating an additive effect. Exposure to either pyraclostrobin or fipronil did not result in significantly different reservoir areas from the other two conditions (Fig. 2).

### Individual and combined effects of pyraclostrobin and fipronil on hypopharyngeal glands

Pesticide exposure did not significantly alter the total number of acini in the hypopharyngeal glands of nurse honeybees (Table 1).

**Table 1:** Total acini count in the hypopharyngeal glands of nurse honeybees.

Treatment	Control	Pyraclostrobin	Fipronil	Pyraclostrobin + Fipronil
Total number of acini	1,230 (1,218–1,340)	1,279 (1,243–1,310)	1,290 (1,267–1,352)	1,290 (1,250–1,319)

Six days after exposure, total acini were counted in serially collected histological sections taken at 30 µm intervals from each honeybee (n = 10). No statistically significant differences were detected (Kruskal–Wallis one-way ANOVA on ranks, p = 0.418). Data are presented as medians and interquartile intervals (Q1–Q3).

**Figure 3:**
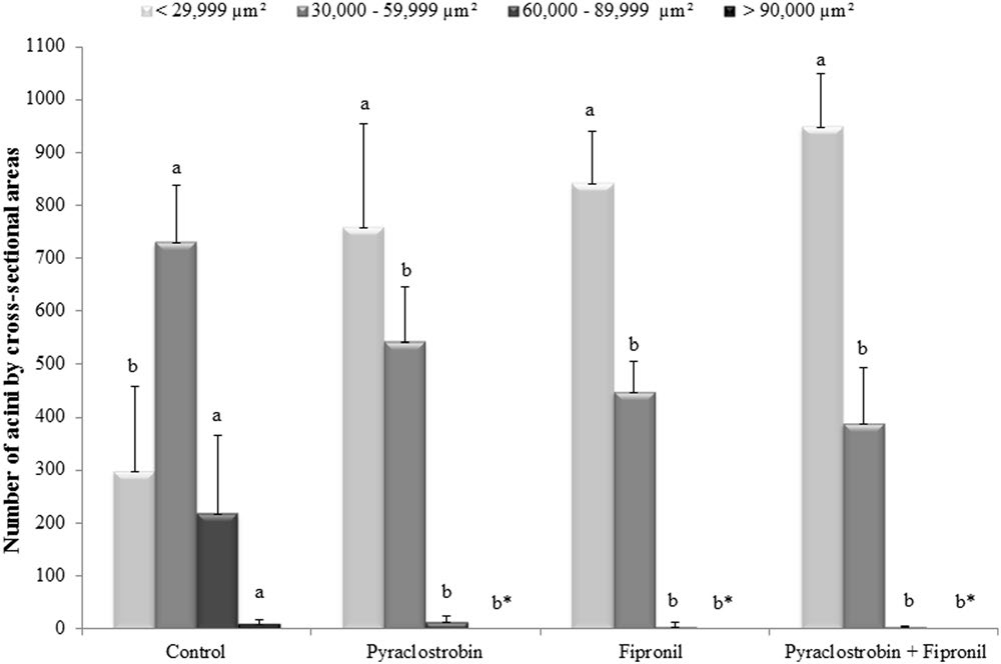
Cross-sectional areas of acini in hypopharyngeal glands measured in nurse honeybees exposed to treatments of pyraclostrobin and fipronil both individually and combined. Acini area was measured in serially collected histological sections taken at 30 µm intervals from each honeybee (n = 10) and then categorized by area. Different letters over bar graphs represent statistically different groups (Kruskal–Wallis one-way ANOVA on ranks with Dunn’s post-hoc test for pairwise comparisons, p < 0.001). All data are represented as the median with interquartile range. *Acini with areas >90,000 µm² were not found.

**Figure 4:**
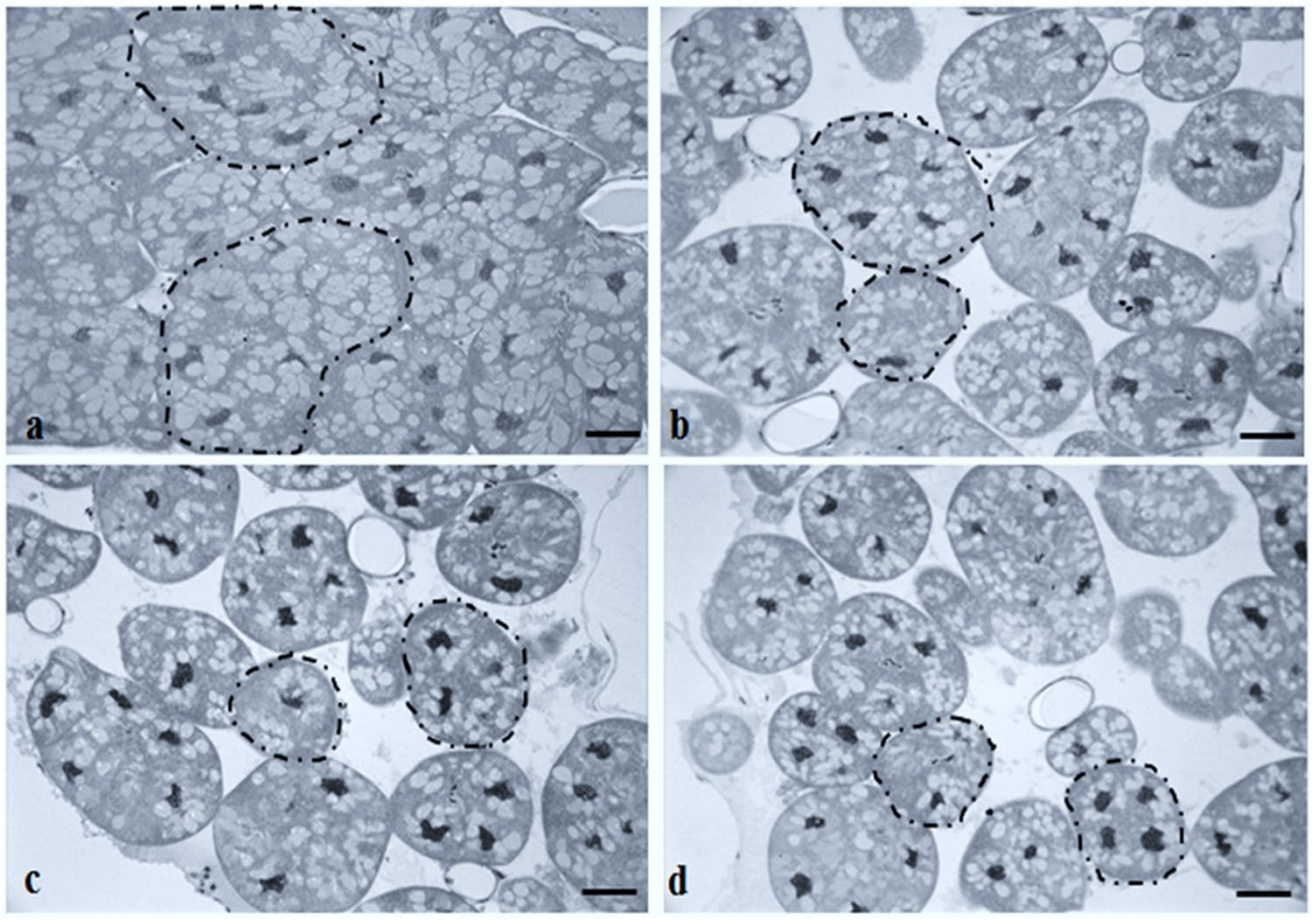
Representative histological sections of hypopharyngeal glands in nurse honeybees. (**a**) Control and (**b**) pyraclostrobin, (**c**) fipronil, and (**d**) pyraclostrobin + fipronil treatments. Note the greater number of acini (emphasized with dotted lines) with reduced area and fewer protein grains in the acini of honeybees exposed to pesticides. Scale bar: 5 µm.

However, when acini were categorized based on area, a more complete view of the changes that occurred could be characterized demonstrating that pesticide-exposed bees had significantly more acini with reduced areas and significantly fewer with larger areas (Kruskal–Wallis one-way ANOVA on ranks, p < 0.001) (Figs 3 and 4). However, the number of acini with reduced areas was not different among pesticide exposure treatments, even when pyraclostrobin and fipronil were combined (Kruskal-Wallis one-way ANOVA on ranks, p > 0.05).

### Colony development

The areas occupied by worker eggs, uncapped brood, and capped brood in the brood frames did not differ between the treatment groups and the control group (Kruskal–Wallis one-way ANOVA on ranks, p > 0.05) (Supplementary Table S1). Mortality of bees inside or around the hives was not observed.

### Chemical residue analysis and validation

Pollen patties supplied to colonies were tested to confirm pyraclostrobin and fipronil dosages. No pesticide residues were found in control patties. The average residue level in patties containing pyraclostrobin was 870 ± 53 ppb (mean ± SD). No fipronil residues were detected in our contaminated patties. We also tested for principal fipronil metabolites (fipronil sulfone, fipronil desulfinyl, fipronil sulfide, and fipronil amide), but none were observed within the limits of detection (LOD = 1.5 ppb). However, given the clear biological changes we observed, fipronil was likely present in pollen patties and had an effect even in concentrations below the LOD.

## Discussion

Stress-related dysfunction in specialized tasks can affect entire honeybee colonies^35^. Here, we demonstrated that exposure to field-relevant doses of pyraclostrobin and fipronil caused alterations in the mandibular and hypopharyngeal glands of nurse honeybees. Specifically, we showed that pesticide exposure reduced epithelial-cell height in mandibular glands, and combined pyraclostrobin + fipronil reduced both cell height and reservoir volume, indicating an additive effect of these pesticides. In hypopharyngeal glands, while the total number of acini (approximately 550 acini/gland according to previous reports^36,38^) was unaffected; more acini had reduced areas in bees exposed to pesticides. However, we observed neither additive nor synergistic effects of pyraclostrobin + fipronil on the development of this gland, likely because of the high reduction of acini area as observed in single-pesticide exposure to fipronil or pyraclostrobin. These data corroborate previous studies showing hypopharyngeal-gland impairment in honeybees exposed to pesticides in the laboratory^35,55–58^ and in bees exposed to pesticides in the field^58,59^. As far as we are aware, our study is the first to examine both the mandibular and hypopharyngeal glands in honeybees exposed to pesticides. Given the importance of these brood-food glands to colony nutrition^41–43,53^, our results newly revealed sub-lethal effects that can affect colony maintenance.

The ratio of mandibular to hypopharyngeal gland secretions deposited around each larva reared in the colony varies depending on larval age, sex, and caste^60,61^. Queen larvae receive only mandibular-gland secretions during the first 3 d, and afterwards receive secretions produced by both the mandibular and hypopharyngeal glands in a 1:1 ratio^60^. Worker larvae are fed secretions from both the mandibular and hypopharyngeal glands in ratios of 1:3 to 1:4^62^, which are mixed with pollen after the third day^63^. Thus, nurse bees without mandibular glands have difficulty rearing queens^64^. Indeed, our work suggests that pesticide-related impairments to both studied glands in nurses resemble the effects of poor nutrition on the colony and brood-food gland development^13,65^. Studies have demonstrated that restricting worker access to nutrients during development causes physical and behavioural problems in adult honeybees^66^. Pesticide-linked alterations to brood-food glands can harm brood care through a reduction in royal jelly quantity/quality, which can threaten caste differentiation, bee development, and colony maintenance, all contributors to high colony losses worldwide^4,67,68^. We highlight that royal jelly production is related to the development of mandibular and hypopharyngeal glands of nurse honeybees^36,38,40,52,53,69^ and emphasize the importance of direct royal jelly measurement in colonies exposed to pesticides in future studies to evaluate its quantity and quality.

Our study provides additional explanations for why exposure to sub-lethal doses of pesticides can reduce colony growth and queen production^18^ while increasing queen supersedure^70^. Queens reared in colonies exposed to pesticides have lower body and ovary weight, if they survive at all^71^. Pesticide studies similar to ours have suggested that compromised immunity in developing queens may be the underlying cause of these symptoms^32^. Here, we hypothesized that nutritional deficits from low-quality/quantity royal jelly produced by nurses with impaired glands would likely contribute to the negative effects of pesticides on queens.

Furthermore, nurse honeybees with hampered hypopharyngeal gland development appear to become foragers more rapidly^56^. Premature foraging activity in honeybees is associated with a reduction in the number of nurses, insufficient brood rearing, and a reduced lifespan, all of which can accelerate colony collapse^58,72^. If this is the case, then the morphological impairments we observed in both the hypopharyngeal and mandibular glands may cause even faster nurse-to-forager changeover, meaning that the colony is fed low-quality royal jelly over a short period.

All honeybees used in our experiments were from the same colonies, reared under the same conditions, and received the same pollen diet. Through the inclusion of pollen and brood pheromones, this experimental design controlled for potential confounding factors that could hamper the full development of brood-food glands even in the pesticide treatments^36,40^. Given that the bees were provided with a generally favourable environment with sufficient food resources, we hypothesize that pesticides reduced nutrient digestion and absorption in nurses, contributing to improper glandular development. Indeed, such effects have been reported in insects^27,28^ with studies demonstrating that exposure to pesticides affect the honeybee midgut^23–25^, including some that demonstrate that fipronil induces death of digestive midgut epithelial cells^26^. Further studies are necessary to explore the connection between nutrition and pesticides exposure and the glandular development of bees in greater detail. In addition, the effects of diet quality on bees exposed to pesticides need be considered, because this factor influences hypopharyngeal gland development in nurse bees according to Renzi *et al*.^57^.

In our study, we focused on two pesticides that can be stored in colonies for long periods and across a range of doses. Our chemical residue analysis of pollen patties confirmed the presence of pyraclostrobin residues within or below the amounts detected in various pollens (mean: 2,787.1 ± 1,890.1 ppb^31^; mean: 10,458 and 267 ppb^49^) and in beebread (range: 319 –2,170 ppb^47^). Thus, our dose of pyraclostrobin substantiated a realistic exposure concentration of this fungicide that bees would encounter. Based on studies that have calculated the total amount of pollen consumed by nurse honeybees^44^, if a nurse bee were to feed on the pyraclostrobin-contaminated pollen patties that were detected in our study (mean 870 ppb), it could consume up to a maximum of 10,440 pg of pyraclostrobin within one day of intensive feeding and a mean total amount of 33,930 pg in six days.

We highlight that the fipronil dose that was chosen for bee exposure in this study is within the range of field-relevant doses of this insecticide that have been detected in pollen (mean: 2.3–2.8 ppb^29,50,51^) and is close to rates broadly authorized for application in fields^50^, which implies that the exposure of bees to this insecticide in the field is similar to the dose used in our study. The dose of fipronil added to pollen patties in our study (2.5 ppb) could result in nurse exposure up to a maximum of 30.0 pg of fipronil within one day of intensive feeding and a total of 97.5 pg in six days, according to one estimate of daily pollen consumption by nurse honeybees^44^. However, observed fipronil residue levels in our patties were lower than we expected. The low field-relevant dose of fipronil that we chose for this study and its probable degradation may have reduced the quantity of fipronil and generated low levels of metabolites that preclude their detection by UHPLC/MS-MS within the LOD in this study. Despite low fipronil concentrations, the contaminated patties caused significant biological alterations in honeybee glands. Thus, residual fipronil and related metabolites were clearly present and had an effect even at doses below the LOD (1.5 ppb) and may be close to the amount of residue detected by Chauzat *et al*.^73^ in pollen (fipronil mean content: 1.2 ppb; metabolite mean content: 1.0–1.7 ppb). The effect of fipronil, even at very low concentrations, impaired the brood-food glands of nurse bees, and similar results have been reported for hypopharyngeal glands by Hatjina *et al*.^56^ and Heylen *et al*.^35^ for bees exposed to the insecticide imidacloprid at sub-lethal doses.

Most studies that found fungicides like pyraclostrobin in pollen and other bee products have not investigated fipronil and their metabolites^31,49^, but primarily included neonicotinoids, which also have deleterious effects on the hypopharyngeal glands of nurse honeybees^51,58^. Mullin *et al*.^11^ detected pyraclostrobin and fipronil in beehives. The highest concentrations of pyraclostrobin detected in wax, pollen, and bees were 438.0, 265.0, and 9.0 ppb, respectively, while mean detection of fipronil in these samples was 0.2, 0.1, and 21.9 ppb, respectively^11^. It is important to highlight the persistence of fipronil and pyraclostrobin in the environment owing to their systemic properties^20,46^. The fact that bees can collect and store contaminated resources in their hives^10–12,20^ and the increase in pesticide detection in migration colonies^51^ can increase bee exposure to pesticides such as those used in our study.

In our study, we choose to work with commercial formulations of pyraclostrobin and fipronil as these are more environmentally relevant and represent a better approximation of the occurrence of bee poisoning in the fields. Some inert ingredients (i.e. builder, preservative, filler, adjuvants, or stabilizer components) can also have toxic effects on honeybees and contribute to the declining health of their populations^74^. A study performed by Ciarlo *et al*.^75^ demonstrated that ingestion of spray adjuvants impairs learning performance of honeybees, and Zhu *et al*.^76^ confirmed that inert ingredients can be highly toxic to developing honey bees; however, we know of no studies comparing the effects of inert ingredients, active ingredients, and commercial formulations of pesticides on mandibular and hypopharyngeal gland development in honeybees. A full-label disclosure of the composition of pesticides, including inert ingredients, is essential^76^ and can enable a more appropriate risk assessment of these substances to pollinators and non-target species^20,74^.

Although a reduction in colony development was not observed in our study, probably because of the short exposure period and low doses of pesticides used, we highlight that impacts that are not detectable at the colony level are still impacting the individual bees. Our results provide evidence that pesticide exposure over a short period (six days) results in clear changes to the mandibular and hypopharyngeal glands of nurse honeybees. Because honeybees can access contaminated and non-contaminated pollen in the field, the deleterious effects may not be as obvious as what we observed. However, honeybees can be exposed to multiple pesticides for a far longer period in the field, with a greater probability of interactions between pesticides. Thus, our results may understate the actual negative impact of pesticide exposure on nurse bees.

Detecting deleterious effects of pesticides in doses that are experienced by pollinators in the field is a critical step toward avoiding the negative impacts that these substances may have on bee maintenance and is crucial in terms of prohibition or re-registration of pesticides. In conclusion, our data highlight the importance of subjecting pesticides to tests that are more rigorous so that sub-lethal effects on important insects like honeybees can be detected. Our results can contribute to the development of policies aimed at promoting more appropriate pesticide risk assessments, mainly at doses close to those that are found in resources collected by bees and to the adoption of management responses aimed at reducing global pesticide application (with emphasis on highly toxic insecticides such as fipronil) to protect pollinators. Because of the negative effects of the fungicide pyraclostrobin on brood food glands, we also highlight the importance of evaluating other molecules of this class that are traditionally considered safe to pollinators and the importance of avoiding spraying blooming crops with them.

## Methods

Test subjects were Africanized *Apis mellifera* kept in the apiary of the Faculty of Veterinary Medicine and Animal Science, UNESP, Botucatu, São Paulo, Brazil, under normal living conditions. All colonies were free of diseases and parasites; thus, no treatment against *Varroa destructor* was performed.

### Experimental design, diet supply, nurse bee collection, and residue analysis

Experiments were performed during March–May 2016. Two brood combs of emerging bees from five donor colonies were removed, marked, and placed in separate, incubated cages under conditions of 34 °C and 80% relative humidity. The thoraces of newly emerged bees (<10 h) of each donor colony were marked with a specific colour using a non-toxic paint (Posca Paint Pens, Mitsubishi Pencil, Japan) to distinguish colony origin. After marking, we randomly distributed approximately 40 bees of each donor colony to the experimental colonies where they received contaminated food.

Each experimental colony was housed in a nucleus with two sealed brood frames, two open brood frames, one egg-laying frame, and approximately 4,000 bees. The queens in each experimental nucleus were sisters, naturally mated and at four months of age. All frames with pollen/beebread were removed from experimental nuclei 24 h before marked bees were introduced. Pollen traps were installed at the entrances to maximize the amount of contaminated diet consumed. Honey syrup (50% w/v) was supplied *ad libitum* to all colonies.

The areas (cm²) occupied by worker eggs, capped brood, and uncapped brood in all frames in the nucleus were evaluated according to Zaluski *et al*.^20^. Measurements were performed at the beginning of the study when food was supplied and every 2 weeks, for a period of 75 days.

All pollen and honey used in this study were organic and polyfloral (Breyer & Cia Ltda, União da Vitória, Brazil) to avoid pesticide cross-contamination. Stock pyraclostrobin and fipronil solutions were prepared from formulated products (Comet^®^ 250 g a.i. L^−1^, inert ingredients 802 g L^−1^, BASF Schwarzheide GmbH, Schwarzheide, Germany; Regent 800WG^®^ 800 g a.i. kg^−1^, inert ingredients 200 g kg^−1^ BASF Agri-Production SAS, Saint Aubin Les Elbeuf, France). Compositions of inert ingredients were not disclosed on the labels for pesticides used in this study. All commercial pesticides were diluted in distilled water. The final concentration of each treatment dose (pyraclostrobin: 850 ppb; fipronil: 2.5 ppb; pyraclostrobin + fipronil: 850 ppb + 2.5 ppb) was obtained through adding concentrated pyraclostrobin and fipronil solutions to honey syrup (50% w/v). This mixture was added to pollen powder in a 3:1 (pollen to honey syrup) ratio. Pollen patties were homogenized, portioned in cellophane paper (100 g), and stored in a freezer (−20 °C) until use. Control pollen patties without pesticides were prepared following the same procedure.

Residue analysis was performed on two subsamples (50 g) of each pollen patty, based on the QuEChERS (quick, easy, cheap, effective, rugged, and safe) pesticide extraction method^77^. The QuEChERS citrate-buffering version (European Standard EN 15662)^78^ was modified based on that of a previous study developed for pesticide and antibiotic residue determination in honey samples^79^. Ultra-high-performance liquid chromatography coupled with tandem mass spectrometry (UHPLC-MS/MS) analysis of the residues was performed at the Laboratory of Pesticide Residue Analysis (LARP), Chemistry Department, Federal University of Santa Maria, Santa Maria, Brazil. Pesticide standard materials with purity >99% were purchased from Dr. Ehrenstorfer (Germany). To ensure quality and comparability of results, pesticide residues in pollen patties were validated according to the methods of the Brazilian Health Surveillance Agency Legislation^80^. Method selectivity was assessed by analysing blank samples and was checked for any interference around the retention time of the target analytes; no interfering compounds were observed by UHPLC-MS/MS. Linearity, calculated based on the spike levels for each analysed compound, resulted in satisfactory calibration curves in the range 0.1–200 μg kg^−1^ with determination coefficients (r^2^) higher than 0.99 for all compounds and a relative standard deviation below 20%.

Chromatographic separation was performed with an Acquity BEH C18 column (50 × 2.1 mm; 1.7 μm particle size) from Waters (Milford, USA) maintained at 45 °C. Mass spectrometric (MS) analyses were performed using the selected reaction monitoring mode. All MS parameters were optimized under the electrospray ionization (=/−) mode. Because fipronil was not detected initially, its principal metabolites (fipronil sulfone, fipronil desulfinyl, fipronil sulfide, and fipronil amide) were analysed. The LOD was 1.5 ppb for pyraclostrobin, fipronil, and all fipronil metabolites.

Each experimental colony received one contaminated pollen patty daily (for 6 d), placed on the top bars of frames, and the amount acquired by the colony was measured by weighing the food not consumed. This measure ensured that marked honeybees consumed contaminated pollen *ad libitum* from the first day until nursing, when they produce more royal jelly^36,38,53,54^. The amount of pollen patties acquired daily by the colonies did not differ between the treatment group and the control with an average daily amount consumed of 80 ± 6 g (mean ± SD) among all colonies (Kruskal–Wallis one-way ANOVA on ranks, p = 0.328). Ten marked nurse honeybees (two per donor colony, distinguished according to their colour) were collected in each experimental colony on day 7 (after 6 d of exposure to the contaminated diet). Bees were anesthetized in a freezer and then decapitated.

### Processing of material for morphological analysis

To increase exoskeleton permeability, entire heads were fixed (4% paraformaldehyde/1 × PBS) for 24 h, washed with distilled water, and transferred to a solution containing sodium hypochlorite (5%) and sodium hydroxide (7.5%) for 5 h at room temperature (25 ± 2 °C). Heads were dehydrated using an ascending ethanol series (70, 80, 90, and 95%; 2 h per bath). Subsequently, a cut was made in the anterior and posterior portions of the heads (eliminating about 1 mm of chitin) under a stereomicroscope. The heads were embedded in resin (Leica Historesin Embedding Kit; Leica, Nussloch, Germany) and sliced with a microtome (Leica RM2155; Germany); frontal cross-sections were 3 μm and separated by 30 µm. Histological sections were made every 30 μm to avoid measuring acini from the same hypopharyngeal gland more than once^69^ and to guarantee evaluation of mandibular glands throughout their extension. Sections were stained with haematoxylin and eosin^24^. Stained sections were examined under a Leica DMLB 80 microscope connected to a Leica DC300FX camera. Digitized images were analysed morphometrically using the bundled software, Leica Q-win version 3.1 for Windows^TM^ (Leica, Heidelberg, Germany).

Epithelial cell height in the mandibular glands did not vary much between experimental groups. Thus, the height of 10 cells was measured for each gland section (n = 10) per bee (n = 10) for a total of 1,000 cell measurements; all glands and cells were randomly selected. Analyses were performed on mean cell height per measured gland. The total area of the mandibular glands (one pair cross-sectioned per animal) was determined from 20 histological sections using 400 measurements in total and then, the glands were classified according their size (<499,000; 500,000–1,000,000; and >1,000,000 µm²) for statistical analyses to evaluate if a change in the area influenced the reservoir volume of these glands.

For the hypopharyngeal glands, the number and area of acini were measured in all histological sections where they were present. Considering that acini area is the main indicator of hypopharyngeal gland activity and royal jelly secretion^36,53,54,57,69^, acini area per bee (n = 10) was categorized by size (<29,999; 30,000–59,999; 60,000–89,999; and >90,000 µm²) for statistical analyses. This standardized histological method to analyse acini area is less influenced by evaluation bias than is visual assessment of acini size (e.g. choice of acini, ability to extract and measure them) and thus, is more appropriate for measuring acini that display an irregular form^57^.

### Statistical analyses

Measurement data were first tested for normality and homogeneity of variance by Kolmogorov–Smirnov and Levene’s test, respectively. Data were compared by Kruskal–Wallis one-way ANOVA on ranks and Dunn’s post-hoc tests for pairwise comparisons of all groups using SigmaStat (version 3.5; Systat Software Inc., San Jose, CA). Significance was set at p < 0.05.

### Data availability

The datasets generated and/or analysed in the current study are available from the corresponding author on reasonable request.

## Electronic supplementary material

Supplementary Table S1
