# Supplementary material for: Field-relevant doses of the systemic insecticide fipronil and fungicide pyraclostrobin impair mandibular and hypopharyngeal glands in nurse honeybees (Apis mellifera)
Source: Sci Rep. 2017 Nov 9;7:15217. doi: 10.1038/s41598-017-15581-5 (PMC5680249; doi:10.1038/s41598-017-15581-5)
Supplement: Supplementary file 1 — Supplementary Table S1 [file 41598_2017_15581_MOESM1_ESM.pdf]

Supplementary Data

Field-relevant doses of the systemic insecticide fipronil and fungicide pyraclostrobin impair mandibular and hypopharyngeal glands in nurse honeybees (*Apis mellifera*)

Rodrigo Zaluski[1], Luis Antonio Justulin Jr.[2], Ricardo de Oliveira Orsi[1,*]

Affiliations:

[1]Núcleo de Ensino, Ciência e Tecnologia em Apicultura Racional (NECTAR), São Paulo State University (UNESP), School of Veterinary Medicine and Animal Science, Department of Animal Production, Botucatu, SP, Brazil

[2]São Paulo State University (UNESP), Institute of Biosciences, Department of Morphology, Botucatu, SP, Brazil

Supplementary Table S1 ………………………………………………………………………………………….……….Page 2

Supplementary Table S1. Areas occupied by worker eggs, uncapped brood, and capped brood (cm²) in combs in honeybee colonies exposed to pyraclostrobin and fipronil, both individually and combined.

Days after exposure	Worker eggs				Uncapped brood				Capped brood			
	**Ct**	**Pyr**	**Fip**	**Fip+Pyr**	**Ct**	**Pyr**	**Fip**	**Fip+Pyr**	**Ct**	**Pyr**	**Fip**	**Fip+Pyr**
**15**	216 (74-228)	212 (71-218)	200 (66-209)	188 (64-196)	312 (108-330)	280 (96-322)	240 (92-296)	220 (86-260)	328 (124-366)	320 (98-355)	300 (64-320)	292 (70-330)
**30**	232 (130-354)	236 (106-320)	220 (86-280)	208 (86-286)	228 (126-284)	220 (106-256)	212 (90-239)	200 (90-216)	212 (88-248)	200 (89-216)	192 (83-206)	166 (71-190)
**45**	212 (118-312)	220 (116-274)	192 (127-254)	144 (106-234)	212 (118-274)	200 (86-285)	192 (105-246)	180 (82-214)	228 (145-358)	216 (124-316)	212 (110-284)	196 (94-292)
**60**	264 (146-356)	236 (106-317)	260 (100-304)	240 (88-278)	284 (172-300)	260 (172-290)	220 (168-270)	212 (142-254)	204 (133-274)	212 (125-276)	196 (115-248)	184 (104-208)
**75**	212 (118-274)	220 (118-276)	204 (108-292)	184 (98-254)	244 (174-318)	224 (166-314)	216 (168-320)	204 (142-302)	232 (138-372)	216 102-336)	240 (96-304)	220 (84-282)

No statistically significant differences were detected (Kruskal–Wallis one-way ANOVA on ranks, $p > 0.05$). Data are presented as medians and interquartile intervals (Q1–Q3). Ct: control; Pyr: pyraclostrobin; Fip: fipronil; Fip+Pyr: fipronil + pyraclostrobin.
